# Challenges in finding and measuring behavioural determinants of childhood obesity in Europe

**DOI:** 10.1007/s10389-015-0657-8

**Published:** 2015-03-01

**Authors:** Denise Alexander, Michael J. Rigby, Pasquale Di Mattia, Anja Zscheppang

**Affiliations:** 1 Department of International Health, School for Public Health and Primary Care (CAPHRI), Maastricht University, PO Box 616, 6200 MD Maastricht, The Netherlands; 2 School of Public Policy and Professional Practice, Chancellor’s Building, Keele University, Keele, Staffordshire ST5 5BG UK; 3 Nordic School of Public Health, Box 12133, 402 42 Gothenburg, Sweden; 4 School of Public Policy and Professional Practice, Chancellor’s Building, Keele University, Keele, Staffordshire ST5 5BG UK; 5 S. Elia Hospital, Caltanissetta, Italy; 6 Centro per la Formazione Permanente e l’Aggiornamento del Personale del Servizio Sanitario (CeFPAS), Caltanissetta, Italy; 7 Research Association Public Health Saxony and Saxony-Anhalt, Technische Universität Dresden, Fiedlerstraße 33, 01307 Dresden, Germany

**Keywords:** Obesity, Childhood, Data, Surveillance, Europe

## Abstract

**Aim:**

Childhood obesity is an important concern for child health. However, despite widespread concern about the increase in childhood obesity, its causes are not monitored systematically in Europe. In 2007, the Scientific Platform Project on Lifestyle Determinants of Obesity identified routine data sources nationally available in European countries to measure childhood obesity. This work was revisited in 2014 to monitor any progress made.

**Subject and methods:**

In 2007, a literature review and project discussion resulted in a list of desirable indicators that could be collected in Europe to describe child populations at risk of overweight and obesity. Participants from EU member states, the EEA, Croatia, Macedonia and Turkey set out to discover which countries collected these indicators. Eight years later, a literature search sought to establish if the surveillance of children’s nutrition and physical activity behaviour had changed.

**Results:**

In 2007, no countries collected all variables for all ages, leading to major gaps in knowledge. A literature search carried out in 2014 suggests that this is unchanged. There remains inconsistency of data surveillance in Europe, and disagreement on which age groups to collect data from or how to define obesity and overweight.

**Conclusion:**

There is a lack of consistent data collection on upstream influences on obesity. The true causes of the childhood obesity epidemic remain undiscovered, and the ability of research to identify effective prevention and treatment methods is compromised.

## Introduction

Childhood obesity is one of the most serious public health problems facing the world today, and has been described by the World Obesity Federation (2012) as a health challenge that is on the verge of imposing unprecedented strain on global health, healthcare, financial capacity and on society as a whole (World Obesity Federation 2012). The rates of childhood obesity have trebled since the 1980s (World Obesity Federation 2012) and it is estimated that in 2010, one in three children aged between 6 and 9 years old were overweight; this is a marked increase in the numbers recorded in 2008, when one in four was overweight (Wijnhoven et al. 2014). Obesity places huge demands on European health systems. It is estimated that European nations spend between 2 and 8 % of their healthcare budgets on diseases that are related to obesity (World Obesity Federation 2012) and unless the epidemic is tackled effectively, this burden will only increase.

Childhood obesity is an important concern for child health and in the context of a healthy ageing agenda. It is a predictor for many adult chronic diseases. Measuring elements of childhood lifestyles and behaviour is particularly challenging, but nevertheless important, because childhood overweight and obesity has the greatest life-long consequences (World Health Organisation 2006), and childhood is a time of vulnerability to micro and macro environments. Recent research trends are to investigate the wider determinants that ultimately result in overweight, such as the influence of the built, social, political and economic environments (Brug et al. 2008; Brug 2008; van der Horst et al. 2007; Kleiser et al. 2009; Owen et al. 2014); however, there are not the national or subnational data on children comprehensive enough to make robust international comparisons or draw conclusions. This state of affairs has resulted in confusing or conflicting evidence surrounding the environment as a factor in obesity (Brug 2008). The Health Behaviour of School-aged Children (HBSC) study, which was, and is, used as an important source of knowledge about the child population in many countries, notes the importance of social context in the development of protective factors and reduction of risk factors in terms of obesity and overweight (Currie et al. 2012); but even the HBSC study report notes how difficult it is to measure physical activity or sedentary behaviour in a way that makes identifying trends in relation to socioeconomic status, for example, easy to understand and to act upon (Currie et al. 2012).

Coordinated international activities are a vital tool in this quest for better practical information for two principal reasons. First, international comparison is a powerful driver for change. Countries in the least favourable positions, having been made aware of this, are more motivated to find the resources and knowledge to effect improvements, but can also see which of their peer nations may be able to share guidance for success. Second, understanding and measuring the determining factors of health-related behaviour in this complex field is highly challenging and, thus, the pooled skills of international agencies and programmes can best assist all concerned.

In 2007, the Scientific Platform Project on Lifestyle Determinants of Obesity, a European Union (EU) funded project, set out to monitor the childhood obesity epidemic. This attempted to determine how and what data were collected nationally by 31 countries from the EU, members of the European Economic Area, Croatia, Macedonia and Turkey. This project was a partner exercise to an exploration of what data were known about adult obesity in Europe (Wolfram et al. 2008). In the 7 years since the completion of the project, and subsequent publication of the accompanying book (Alexander et al. 2010), childhood obesity has become an increasingly important public health issue with implications for current and future population health.

This paper describes the findings of the original project and investigates the progress, if any, that has taken place since 2007 in collecting data on the childhood obesity epidemic. Accurately monitoring prevention efforts as well as the prevalence of the epidemic in Europe is essential to reduce childhood obesity. This is because children are arguably the section of the population most influenced by environmental and societal determinants of behaviour; they are largely dependent on adults for their health and wellbeing. Any population surveillance approach must reflect this and not only identify the prevalence of overweight, but also the prevalence of ‘upstream’ factors that increase the risk of overweight such as lack of availability of fresh food, or no safe places for physical activity.

## Methods

Four scientific partners collaborated in the original Lifestyle Determinants project, and twenty country-specific sub-contractors gathered data from all 31 countries in the project. Its purpose was to identify routine data sources that were available nationally in Europe. The definition of ‘routine data source’ was that all people can access the information; and that data are regularly and periodically gathered, for example for legal, operational, or reimbursement purposes such as vital statistics, census data, hospital care statistics, or ambulatory care statistics.

In the 2007 project, a literature search was conducted to determine the type of data that could be sought. Peer-reviewed literature relating to children’s nutrition and physical activity was identified through PubMed. Search terms for this literature were specific for each age group and for the type of behaviour under investigation—for example: ‘infants’ and ‘nutrition behaviour’; ‘adolescents’ and ‘physical activity behaviour’; ‘obesity’ and ‘children’; ‘obesity’ and ‘5–9 year olds’. In total, 27 separate searches were undertaken. These searches were supplemented by a follow up of references and consultation with identified experts in the fields of nutrition, physical activity and obesity. Relevant topics and indicators were refined into formal data definitions during a 2-day workshop by the four main project partners from Germany, Italy, Sweden and the United Kingdom. It became obvious that solely updating the adult-oriented project’s database of routine national surveys and data sources to include those relating to children would not give national policy makers adequate evidence of behavioural determinants for all child life-course stages. For the child-focused project, therefore, key indicator sets were created separately for nutrition and physical activity for each main stage of childhood. A child was defined according to the United Nations Convention on the Rights of the Child (United Nations Office of the High Commissioner for Human Rights 1989), namely, persons from birth up to their 18th birthday. Indicators were sought for 5-year age bands; and/or pragmatic school-based age bands reflecting children’s developmental stages.

Once the parameters were established, data collection began. Each country provided a one-page country profile detailing the priority given to childhood obesity by government, health services and mass media; completed the two agreed schedules of data availability; and updated the pre-existing adult related project database regarding projects with children within the included age groups, adding information about age groups, gender, data collected and the availability of data on behaviour measures. The national subcontractors were asked to find information readily available to them, or at least to be found with minimal effort, to replicate what an ‘informed layperson’ could achieve.

In 2014, the original literature review was repeated as far as possible. PubMed was interrogated to find peer-reviewed papers on childhood nutrition, physical activity and measurement. The search terms were necessarily general in order to identify as wide a range of articles as possible. They were: (‘child’ or ‘adolescent’) and (‘nutrition behavio*’ or ‘physical activity behavio*’) and ‘health surveillance’; both US and UK English spellings were used for the term ‘behaviour’. In addition, a search of the EU and WHO websites was undertaken for relevant publications, and references of all papers and reports were followed up. Fifteen research papers, position papers and policy documents that had been published since 2007 on the subject of national surveillance of childhood obesity and overweight in the countries in Europe were read.

## Results

The results of the 2007 Lifestyle Determinants project and the 2014 update exercise will be presented here in turn. The Lifestyle Determinants project (2007) enabled an overview of the pattern of data availability, and identified major gaps in surveillance of upstream determinants of nutrition and physical activity among children by topic or by country. The results showed whether the data were available in each country, and to some extent how easy the data were to retrieve.

Two data types were included in the final indicators identified and chosen for the project database in 2007: statistical data (of which there were 39 indicators) and policy and legal instruments (of which there were 37 indicators). The latter are potentially powerful in influencing behaviour modification, and so logically needed to be included (World Health Organisation 2013; Brug et al. 2008). The most desirable data sources were those from country-level routine and repeated data collection; but the study also included robust surveys, such as the HBSC study (Currie et al. 2012), one-off statistical activities and published research studies. Most of the indicators were of salutogenic or protective behaviours which have correlations with positive health, although a few were of sedentary behaviour, which may indicate a heightened risk of obesity. There was a reasonably even spread of indicators across all age groups in terms of statistical and legal and policy items.

The next and central component of the Lifestyle Determinants project was to identify the degree to which the country subcontractors could feasibly obtain these statistical measures from published data sources, and establish the existence of the Legal and Policy mechanisms. Table 1 shows how successful they were in terms of statistical items and shows where data were available from repeated and routinely analysed sources. In addition, some countries reported data availability from one-off research sources, or reported that raw data were available but not routinely analysed and published. Further details are available in the full project report (Alexander et al. 2010). The countries that were identified as collecting data for nearly all of the indicators and the required age groups used a combination of routine data sources such as national surveys or longitudinal studies; data that was collected as part of a routine source, but that is not normally analysed in a child-focused way; and research and one-off studies. Routine sources are preferable in order to monitor an epidemic; but are rarely available in a child-focused and upstream format. The potential of existing data that is not currently analysed in a child-focused manner is recognised, but these are initially costly and difficult data to obtain. Research and one-off studies are essential for science, but of limited use in monitoring a long-term epidemic.

**Table 1:** Reported availability of data about population statistical indicators (2007 data)

	Nutrition behaviour: statistical indicators (reported data availability)						Physical activity behaviour: statistical indicators (reported data availability)					
	Birth to 1 Year	1–4 years	5–9 years	10–14 years	15–17 years	% available	Birth to 1 Year	1–4 years	5–9 years	10–14 years	15–17 years	% available
No. of indicators	4	3	3	5	5		0	0	6	7	7	
Austria			3	5	5	65.0			3	5	4	60.0
Belgium	3	1	2	5	5	80.0			5		5	50.0
Bulgaria	1			1		10.0				1	1	10.0
Cyprus		1				5.0						0.0
Czech Republic			2	5	5	60.0				5	6	55.0
Denmark				5	5	50.0				6	6	60.0
Estonia				5	2	35.0				5	5	50.0
Finland		3	3	5	5	80.0			6	6	6	90.0
France	3	3	3	5	5	95.0			6	6	6	90.0
Germany		1				5.0						0.0
Greece	4			4	5	5.0				5	5	50.0
Ireland	1			5	5	52.4				5	5	50.0
Italy	2	1	3	4	5	71.4			5	5	6	80.0
Latvia						0.0				5		25.0
Liechtenstein						0.0						0.0
Lithuania	4			5	5	66.7					5	25.0
Luxembourg	1			5	5	52.4				6	5	55.0
Macedonia	4	3	3	4	3	81.0			2	2		20.0
Malta				5	5	47.6				5	5	50.0
Netherlands	2	1	3	5	5	76.2			3	3	4	50.0
Norway	3			5	5	61.9				5	6	55.0
Poland						0.0						0.0
Portugal						0.0						0.0
Romania	3		2	5	4	66.7			5	4	5	70.0
Slovakia		1				4.8						0.0
Spain			2		2	19.0			5			25.0
Sweden	3	2	2	5	5	81.0				6	6	60.0
Turkey	2					9.5						0.0
UK	4	2	3	5	5	90.5			6	7	7	100.0
Average	1.3	0.6	1.0	3.1	3.0				1.5	3.1	3.3	
%	33.3	21.1	34.4	62.0	60.7	45.7			25.6	43.8	46.7	39.3

Table 1 shows that, in 2007, nutritional behaviour was better monitored than physical activity, and that there was greater availability of data about older children for both nutrition and physical activity. National availability fluctuated considerably but the positive influence of specific coordinated European Studies was evident.

Table 2 shows the identifiable existence of the Legal and Policy items by country in 2007. Data about infant feeding policies were the most readily available, followed by those regarding physical activity for children under 10 years of age. There was much less knowledge of the existence of policy and legal controls for children aged over 10. National policy existence ranged widely between countries, and there were marked differences between the nutritional and physical activity items.

**Table 2:** Reported availability of data about desirable legal and policy actions (2007 data)

	Nutrition behaviour: legal and policy indicators (reported data availability)						Physical activity behaviour: legal and policy indicators (reported data availability)					
	Birth to 1 year	1–4 years	5–9 years	10–14 years	15–17 years	% available	Birth to 1 year	1–4 years	5–9 years	10–14 years	15–17 years	% available
No. of Indicators	3	4	6	4	5		0	1	2	6	6	
Austria	2		1		1	18.2			1	3	1	33.3
Belgium	3		2			22.7		1	1	1	1	26.7
Bulgaria	3	1	1	2		31.8		1	2	4	4	73.3
Croatia	2	3	1	2	1	40.9		0	2			13.3
Cyprus	3	0	2	1	1	31.8		0	2	3	4	60.0
Czech Republic	3	3	2	2	3	59.1		0	2	4	4	66.7
Denmark	2					9.1			1	1	1	20.0
Estonia	3	3	4	4	3	77.3		1	2	1	1	33.3
Finland	3		6	3	3	68.2						0.0
France	3	3	4	2	3	68.2		1	1	1	1	26.7
Germany	3		0		1	18.2			2			13.3
Greece	2	1	0	0		13.6		1	1	3	3	53.3
Ireland	3	0	5			36.4			1		1	13.3
Italy	3		0			13.6		1	1	1	1	26.7
Latvia	3		4	4	5	72.7		1	1		2	26.7
Liechtenstein	2	0	3	0	0	22.7		1	2	3	3	60.0
Lithuania	2	4				27.3		1	1	2		26.7
Luxembourg	2		0			9.1		0	0			0.0
Macedonia	3	0	3	0	0	27.3		1	2	2	1	40.0
Malta	0		0			0.0		0	0			0.0
Netherlands	2	0	1	0	1	18.2			1			6.7
Norway	3		1	2	2	36.4			2	1	1	26.7
Poland	3	0	0		0	13.6		0	1	1	1	20.0
Portugal						0.0		0	1			6.7
Romania	2	4	6			54.5		1	1	1	2	33.3
Slovenia	3	4	2	1	1	50.0		1	1	3	3	53.3
Spain	3	0	2	0	0	22.7		0	0	1	1	13.3
Sweden	3	4	6	3	5	95.5		1	1	2		26.7
Turkey	3	1	1			22.7		0	1	1	1	20.0
UK	3		6	3	4	72.7		1	1	2	2	40.0
Average	2.5	1.0	2.1	1.0	1.1			0.5	1.2	1.4	1.3	
%	83.3	25.8	35.0	24.2	22.7	35.2		46.7	58.3	22.8	21.7	28.7

The Lifestyle Determinants project of 2007 was followed up in 2014. The update of the project was limited by budget and time constraints, meaning that a repeat of the original country-based exercise was not possible. However, current literature and lack of evidence of changes to statistical systems support the conclusion that little has changed, and work needs to be done to improve surveillance of behavioural determinants of health in Europe. From the 15 relevant papers retrieved in the literature search, a picture emerges of a still incomplete profile of the aetiology of childhood obesity. A recent exercise by the World Health Organisation (WHO) aimed to collect data on selected surveillance indicators and policy items for adults, adolescents and children in a similar vein to that carried out by the Scientific Platform project (World Health Organisation 2013). To identify the number of overweight and obese adolescents, they used data from the HBSC study, as did many of the countries in the Lifestyle Determinants project. However, this only gave comparable data for 33 out of the 53 countries in the WHO exercise. The prevalence of overweight in other countries was given for ages 15–19, but the data used the adult Body Mass Index (BMI) calculation criteria, which may not be fully appropriate for children, and so the results cannot be compared with the HBSC countries, and must be used with caution. When describing children aged between 5 and 9 years, data came from the European Childhood Obesity Surveillance Initiative (COSI; World Health Organisation 2007), and only 12 states out of 52 were able to produce data of how many children were overweight in any comparable way. Data for other countries was included when it could be found, but the results cannot be compared internationally. Data for breastfeeding—an indicator proposed as important by the Lifestyle Determinants project and the WHO exercise—produced even fewer results. No inter-country comparable data source could be identified by WHO in 2013, so nationally identified survey data had to be used to estimate the prevalence of breastfeeding; and it is impossible to create a continent-wide picture of breastfeeding prevalence (World Health Organisation 2013). A review in 2010 found very few data relating to pre-school children’s dietary habits and physical activity in relation to overweight. In their search of the global database on child growth and malnutrition, the authors found that 9 out of 27 countries had no nationally representative data on pre-school children (Cattaneo et al. 2010). Similarly, the 2013 Cochrane Review into preventing childhood obesity could only find 55 studies worldwide that were robust enough to form part of their evidence. Within the Cochrane Review, it was found that there was unexplained heterogeneity in the results and also a need for cautious interpretation because of the small scale of many of the studies, which may lead to bias (Waters et al. 2011).

## Discussion

The Lifestyle Determinants project identified feasible ways of measuring upstream determinants of childhood obesity and overweight. Yet, despite the justified expressions of concern about the exponential increase in childhood obesity and calls for action, in 2014 it remains impossible to quantify adequately the countries where children are at most risk of becoming overweight. In 2007, a large disparity in the type and quality of data identified was found, and this remains the case 7 years later, which is somewhat disappointing. The original project showed a considerable variation in policies and in the availability of important behavioural measures through routine statistical sources. No single country provided a template of perfection, but a more robust health-behaviour-monitoring baseline would be achieved if all countries collected the same data as those with more comprehensive health surveillance. The data collection schemes proposed by the Lifestyle Determinants project provide a feasible method of understanding the origins of the childhood obesity epidemic; it is effectively the lack of country systems and motivation that prevents greater knowledge.

The original project gathered a range of indicators, hypothesising that it is the combination of the identified indicators that will give a picture of the behavioural factors that influence the weight of Europe’s school children. Any individual indicator cannot give a true picture of such a complex issue. Most children’s lives feature a combination of healthy and unhealthy behaviours, which can provoke a cumulative effect on overweight and obesity (Leech et al. 2014). There is still a great deal to be discovered, as research has identified only weak associations between diet and physical activity behaviours and obesity, suggesting that effective prevention is more about encouraging an accumulation of small individual changes and a whole lifestyle approach, rather than tackling a single, underlying cause (Jago et al. 2009). As part of such a holistic approach, recent work has identified the important role of sleep, and sleep behaviour patterns in the presence of childhood overweight and obesity (Thivel et al. 2015; Adamo et al. 2013). There is also work that suggests important influences, at certain childhood ages, of sleep and leptin production, a hormone that affects appetite (Boeke et al 2014). In addition, sleep apnoea is more common in obese children, a condition that interrupts sleep and may establish a vicious circle that perpetuates overeating, lack of physical activity and increased obesity (Alonso-Álvarez et al. 2014). There is a need for regular data collection that can show nutrition and physical activity actions as part of wider lifestyle behaviours in each country, extending beyond identifying children at risk but also potentially providing valuable raw, comparable data for researchers to understand more about the links between these complicated factors of obesity.

In the Lifestyle Determinants project, there were not enough data to describe children in each life stage collected routinely across Europe. Some countries had no nutrition data for all age bands. In other countries, nutrition data were only available for children aged up to 1 year, or to age 4. The lack of data about breastfeeding was of particular concern. Baby friendly hospitals, where breastfeeding is supported and made easier for the mothers, were an agreed priority by all health ministers (Alexander et al. 2010), yet in the original project and at present, few countries could identify how many baby friendly hospitals they had. This can be seen as perhaps an indicator of a greater emphasis on rhetoric than on consistently applied simple measures such as scientifically validated data collection. For all age groups and in all countries, there was a comprehensive dearth of information about physical activity. However, the indicators about television watching and time using computers are collected by almost all European countries, for most age groups, even though this is only a weak indicator. In the Lifestyle Determinants project, the majority of countries in Europe collected some of the indicators but not for all age groups. In addition, it is difficult to establish the reliability of some of the data sources, and the inclusion of one-off projects means that some countries may have collected data on a section of the child population but have not repeated the exercise to assess a trend.

A limitation of the original project was that the quality and comparability of the data collected could not be ascertained confidently because the project database did not contain the actual data collected by the individual countries, just the measures or indicators used. However, definitions of measures in use showed that many were unable to be compared between countries, and this remains the case as evidenced by current research (Cattaneo et al. 2010). Incomparability of data between countries in Europe remains a problem in 2014. Many data are incompatible with other country data because of different methods of calculation (Cattaneo et al. 2010). Some countries use, for example, WHO charts for ages 5–19 (de Onis et al. 2007); and others use those of the Centers for Disease Control (CDC; Kuczmarski et al. 2002) or the International Obesity Task Force (IOTF) definitions (Cole et al. 2000) as a basis for their calculations. Each system uses different reference populations and cut offs, making vital international comparisons extremely challenging, if not impossible (Cattaneo et al. 2010). The National Child Measurement Programme in England weighs and measures all children at the beginning and end of state primary school (Lifestyle Statistics Team, Health and Social Care Information Centre 2013), giving a prevalence of underweight, healthy weight and overweight children; but the scheme does not give an indication as to root causes. The European Childhood Obesity Surveillance Initiative aims to collect internationally comparable data on the prevalence of childhood obesity, but does not collect from all EU countries; and does not collect upstream data to explain why certain countries have greater incidence and prevalence of childhood obesity (World Health Organisation 2007). Other important measures, such as the HBSC study, are based on sample populations and not collected routinely every year, and not carried out in all countries of the EU.

The Lifestyle Determinants project had significant implications, necessitating an approach that considers aspects of childhood obesity that ultimately fell beyond its resources. Much of the current effort is placed on simply measuring the number of overweight or obese children in Europe; however, this will only identify effects after they happen. It is important to identify where children may be at more risk of becoming overweight. Lifestyle actions depend on individual choices, including cultural influences, food preferences, time for games, sleep and so on; as well as socioeconomic and environmental factors (affordability of food products and facilities for play and exercise, for example), which are themselves shaped by policies that are the responsibility of the EU Member States and the European Community (EC). Developing a health information system that is sympathetic to these individual choices as well as applicable to the child population is a complex task. At present, the fundamental influences on childhood obesity are not monitored systematically in the EU, even though the means of doing so is possible. The original project broke new ground in identifying desirable scientifically based and internationally comparable measures and indicators. In conjunction with its country-specific subcontractors, it provided a snapshot of both routine data collection and one-off sources of data to achieve these measures. The results were two-fold—a demonstration of the need for further work to formally agree on an indicator set with related data definitions; and a highlighting of the current serious lack of data to illuminate a highly important child public health problem. This is still a worthy conclusion in 2014, as it seems that there are still large gaps in our knowledge about what causes childhood obesity, and how it can be prevented and treated as evidenced by the World Health Organisation exercise among others (World Health Organisation 2013).

The importance of adequate and comparable surveillance systems to inform research and successful prevention and intervention initiatives cannot be underestimated. In order to prevent this status quo from continuing, European nations need to place a greater emphasis on the health surveillance of its children and young people, who make up around of fifth of the European population. Firstly, central agreement on the key data definitions would enable greater comparable data collection and analysis. In addition, developing and agreeing on a formal set of monitoring indicators, not just of overweight and obesity, but of behavioural determinants of nutrition and physical activity in children through all stages of the life course, would provide some clarity about obesity preventing or promoting behaviours that may be taking place. Compiling statistics to these indicators and publishing national data sets would allow focused prevention actions to take place, as well as identifying geographical areas where obesity is a particular risk to the population of children and young people. Alongside these actions, further analysis of the scientific knowledge on the behavioural determinants of nutrition and physical activity behaviour of children and young people is needed. Finally, a compilation of a compendium of effective good practices influencing children’s nutrition and physical activity, as has already been achieved by projects related to children’s environmental exposure and to enhancement of children’s safety, would help to reduce the prevalence of childhood obesity in Europe.
